# Out-of-hospital cardiac arrest: the prospect of E-CPR in the Maastricht region

**DOI:** 10.1007/s12471-015-0782-6

**Published:** 2016-01-04

**Authors:** A.S. Sharma, R.W.M. Pijls, P.W. Weerwind, T.S.R. Delnoij, W.C. de Jong, A.P.M. Gorgels, J.G. Maessen

**Affiliations:** 1 Department of Cardiothoracic Surgery, Maastricht University Medical Center, P. Debyelaan 25, 6202 AZ Maastricht, The Netherlands; 2 Department of Cardiology, Maastricht University Medical Centre, Maastricht, The Netherlands; 3 Departments of Cardiology and Intensive Care, Maastricht University Medical Centre, Maastricht, The Netherlands; 4 Department of Transplantation, Cardiovascular Research Institute Maastricht, Maastricht University Medical Centre, Maastricht, The Netherlands

**Keywords:** Out-of-hospital cardiac arrest, Resuscitation, Shockable rhythm, Return of spontaneous circulation, Survivors, Extracorporeal cardiopulmonary resuscitation

## Abstract

**Aim:**

The current outcome of out-of-hospital cardiac arrest (OHCA) patients in the Maastricht region was analysed with the prospect of implementing extracorporeal cardiopulmonary resuscitation (E-CPR).

**Methods:**

A retrospective analysis of adult patients who were resuscitated for OHCA during a 24-month period was performed.

**Results:**

195 patients (age 66 [57–75] years, 82 % male) were resuscitated for OHCA by the emergency medical services and survived to admission at the emergency department. Survival to hospital discharge was 46.2 %. Notable differences between non-survivors and survivors were observed and included: age (70 [58–79] years) vs. (63 [55–72] years, *p* = 0.01), chronic heart failure (18 vs. 7 %, *p* = 0.02), shockable rhythm (67 vs. 99 %, *p* < 0.01), and return of spontaneous circulation (ROSC) at departure from the site of the arrest (46 vs. 99 %, *p* < 0.01) and on arrival to the emergency department (43 vs. 98 %, *p* < 0.01), respectively. Acute coronary syndrome was diagnosed in 32 % of non-survivors vs. 59 % among survivors, *p* < 0.01. Therapeutic hypothermia was provided in non-survivors (20 %) vs. survivors (43 %), *p* < 0.01. Percutaneous coronary intervention (PCI) was performed in 14 % of non-survivors while 52 % of survivors received PCI (*p* < 0.01). No statistical significance was observed in terms of gender, witnessed arrest, bystander CPR, or automated external defibrillator deployed among the cohort. At hospital discharge, moderately severe neurological disability was present in six survivors.

**Conclusion:**

These observations are compatible with the notion that a shockable rhythm, ROSC, and post-arrest care improve survival outcome. Potentially, initiating E-CPR in the resuscitation phase in patients with a shockable rhythm and no ROSC might serve as a bridge to definite treatment and improve survival outcome.

## Introduction

Out-of-hospital cardiac arrest (OHCA) in developed communities occurs more commonly and is a leading cause of death [1, 2]. Recently, Chan et al. reported a survival rate of 8.6 % in the United States [3]. In the Netherlands, reported survival rates are much higher, from 9 % even up to 43 % [4, 5]. Importantly, determinants that effectively favour better survival outcome include witnessed arrest, bystander cardiopulmonary resuscitation, shockable rhythm, use of automated electrical defibrillator, early return of spontaneous circulation (ROSC) and post-arrest care [2, 6–8]. Collectively, minimising time delays, resuscitation quality, intensive care, and treatment of the underlying cause of arrest increases survival outcome.

Alternatively, extracorporeal cardiopulmonary resuscitation (E-CPR) has been reported to improve survival outcome and organ recovery in refractory cardiac arrest [9–11]. Moreover, expansions in extracorporeal technology such as portable devices, circuitry and access to systemic circulation have led to further curiosity of its potential application [12]. E-CPR application is reported to increase the chances of successful defibrillation, preventing re-arrest due to post-resuscitation myocardial dysfunction, thereby enabling subsequent interventions [11, 13]. Nevertheless, E-CPR requires minimalisation of time delay to establish qualitative invasive CPR, and establish as a bridge to specific diagnostics and intervention.

Therefore, we aimed to analyse the current outcome of adult patients with OHCA following resuscitation in the Maastricht region with the prospects of implementing E-CPR in the near future.

## Methods

The observational study included adult OHCA patients who survived to emergency department admission at Maastricht University Medical Center, which lies in the southern part of the Dutch province of Limburg, between March 2012 and April 2014. The area is 203 km^2^ in size and has approximately 183,000 inhabitants (Statistics Netherlands 2013, www.cbs.nl). The Maastricht area is served by a university medical centre (Maastricht University Medical Center) and a network of emergency medical services (EMS). The local medical ethics review board approved the study with a waiver for obtaining the informed consent.

A call to the emergency number (112) alerts the regional EMS and other trained first responders including civilian SMS-alert responders and police personnel, equipped with an automatic external defibrillator (AED) in case of suspected OHCA. Two ambulances are dispatched, along with the first responder to perform basic life support. The EMS personnel provide standardised, mechanical CPR with an auto-pulse device (Zoll®, Chelmsford, MA USA) and advanced life support.

### Data collection

The data were collected by a researcher, who accessed the data collected and stored by the ambulance services, and by going through the electronic drive forms daily for resuscitations. The data gathered included Utstein-based recommendations on a form [14], while the ECG strips of the patients were attached to the drive forms of the patient. In case of AED use, the information on the AED application was retrieved from the AED (Corpuls^3^, GS Elektromed, Kaufering, Germany) and sent via an email to the researcher. When the SMS alert responder had provided basic life support, pertinent data were sent to the researcher, which were compiled and verified with the patient records. Pre-existing cardiac risk factors, cardiac comorbidities and past cardiac interventions were retrieved from the hospital records. Data on post-resuscitation management (diagnosis, therapeutic hypothermia or treatment such as percutaneous coronary interventions (PCI), coronary artery bypass graft, implantable cardioverter defibrillator (ICD) or pacemaker) were obtained from the hospital records. Survival was assessed at three time points, i.e. survival to emergency department, survival to hospital admission, and survival to hospital discharge.

### Definitions

Arrests assumed to be of cardiac origin were included. Individuals with a do-not-resuscitate status, with signs of prolonged death and cardiac arrest of non-cardiac origin (for e.g. drowning, exsanguination), were excluded. The ECGs analysed by AED or taken by EMS paramedics were designated as the initial cardiac rhythm. The rhythms were classified as shockable (ventricular fibrillation, ventricular tachycardia), non-shockable (asystole, pulseless electrical activity) and non-threatening (sinus bradycardia, sinus tachycardia or sinus rhythm). ROSC was recorded at the time of departure from the site of the arrest and arrival at the emergency department. Neurological outcome to determine brain injury was scored by charting the modified Rankin Scale instead of the standard cerebral performance score to clearly distinguish mild to moderate neurological disability. The scoring was performed by assessing patient records at the time of discharge and at 1 month, 3 months, 6 months and 1 year follow-up. The modified Rankin Scale scores are allocated as: 0—no symptoms, 1—no significant disability, 2—slight disability, 3—moderate disability, 4—moderately severe disability, 5—severe disability, 6—dead [15].

### Statistical analysis

Data were subjected to normality distribution, and accordingly continuous data are presented as medians [interquartile range] and/or means ± standard deviation. Comparisons between two groups of continuous variables were performed using the Mann-Whitney *U*-test. Categorical variables were compared using Chi-square test with Fisher’s exact test and expressed as percentages. Analyses were two-sided and differences with *p*-values < 0.05 were considered statistically significant. The analyses were carried out using the Statistical Package for Social Sciences version 21.0 (IBM Corp., Armonk, NY, USA).

## Results

### Patients

**Fig. 1:**
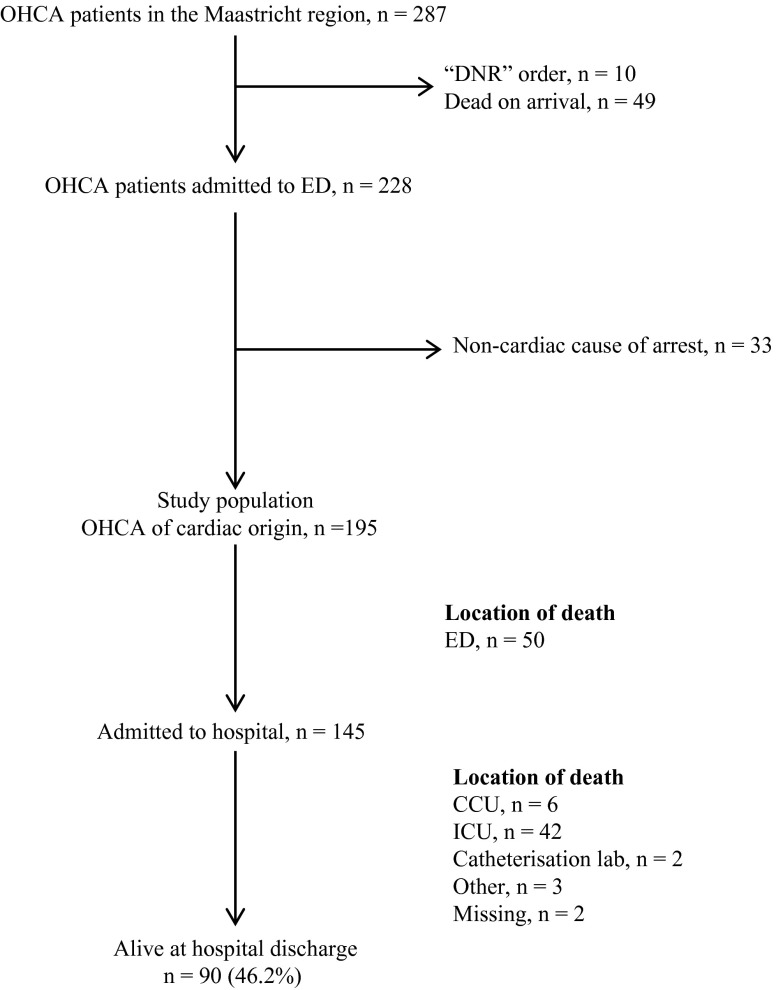
Flow chart of patients with out-of-hospital cardiac arrest in Maastricht region (March 2012–April 2014). *OHCA* out-of-hospital cardiac arrest, *DNR* do not resuscitate, *ED* emergency department, *CCU* coronary care unit, *ICU* intensive care unit; other, ward

A total number of 287 adult patients were identified with OHCA in the Maastricht region through the study period. After assessment of the records, 92 patients were excluded from the study (Fig. 1).

The study population included 195 patients, admitted to the emergency department with a cardiac cause of arrest. Patient demographics (Table 1) and pre-hospital resuscitation characteristics of OHCA are shown in Table 2. The patients were aged 66 [57–75] years and predominantly (80 %) male. The cardiac arrest event occurred at home (56 %) and was witnessed in 173 (89 %) patients. Subsequently 145 (74 %) patients received bystander CPR, EMS personnel initiated CPR in 26 %, and in 24 % cases an AED was applied. Shockable rhythm was observed in 150 (77 %) patients. The EMS response time was 7 [5–9] min, the advanced cardiac life support was provided for 24 [18–31] min, and at departure ROSC was achieved in 70 % of the patients. Transport time to the emergency department was 12 [6–18] min and 61 (58 %) patients required continued resuscitation during transportation.

**Table 1:** Patient demographics of out-of-hospital cardiac arrest of cardiac origin who survived to emergency department admission

	Survivors *n* = 90	Non-survivors *n* = 105	*p*-value^a^
Age (years)			0.01
Mean ± standard deviation	63 ± 12	67 ± 15	
Median [interquartile range]	63 [55–72]	70 [58–79]	
Male gender, *n* (%)	74 (82)	83 (79)	0.57
Cardiac risk factors, *n* (%)			
Diabetes	12 (14)	22 (22)	0.11
Hypertension	29 (34)	35 (37)	0.66
Dyslipidaemia Previous cardiovascular conditions, *n* (%)	14 (17)	18 (19)	0.64
Cerebrovascular accident	4 (5)	6 (6)	0.67
Chronic heart failure	6 (7)	18 (18)	0.02
Myocardial infarction	22 (25)	33 (32)	0.25
Ventricular fibrillation	3 (3)	4 (4)	0.84
Atrial fibrillation Previous cardiac interventions, *n* (%)	7 (8)	17 (17)	0.07
PCI	6 (7)	14 (14)	0.12
CABG	8 (9)	8 (8)	0.78
Pacemaker	2 (2)	0 (0)	0.13
ICD	1 (1)	8 (8)	0.03

*PCI* percutaneous coronary intervention, *CABG* coronary artery bypass grafting, *ICD* implantable cardioverter defibrillator.

^a^Fisher’s exact or Chi-square test for categorical variables; Mann-Whitney *U* test for continous variables.

**Table 2:** Pre-hospital resuscitation characteristics, aetiology and treatment of out-of-hospital cardiac arrest of cardiac origin who survived to emergency department admission

	Survivors *n* = 90	Non-survivors *n* = 105	*p*-value^a^
Location of arrest, *n* (%)			0.79
Home	52 (59)	60 (57)	
Public	36 (41)	45 (43)	
Witnessed arrest, *n* (%)	83 (92)	90 (86)	0.15
Bystander CPR, *n* (%)	68 (76)	77 (73)	0.72
AED deployed, *n* (%)	22 (24)	24 (23)	0.80
Initial rhythm, *n* (%)			< 0.01
Shockable	81 (90)	69 (67)	
Non-shockable	5 (6)	31 (30)	
Non-threatening^b^	4 (4)	3 (3)	
EMS arrival time since call, min			0.36
Mean ± standard deviation	7 ± 6	8 ± 9	
Median [interquartile range]	6 [5–9]	6 [4–9]	
Defibrillation times, *n* (%)			< 0.01
Mean ± standard deviation	1.8 ± 2.0	2.7 ± 2.9	
Median [interquartile range]	1 [0–3]	2 [0–4]	
Auto-pulse, *n* (%)	44 (49)	77 (73)	< 0.01
ROSC at departure, *n* (%)	89 (99)	48 (46)	< 0.01
Continued resuscitation, *n* (%)	1 (1)	57 (54)	
ACLS duration (min), *n* (%)			< 0.01
Mean ± standard deviation	22 ± 8	23 ± 11	
Median [interquartile range]	23 [18–27]	24 [16–31]	
Intubation, *n* (%)	44 (49)	81 (77)	< 0.01
Transport time, min			
Mean ± standard deviation	17 ± 14	16 ± 14	0.36
Median [interquartile range]	15 [9–22]	12 [7–19]	
ROSC on arrival, *n* (%)	88 (98)	45 (43)	< 0.01
Continued resuscitation, *n* (%)	2 (2)	59 (56)	
Cardiac arrest aetiology, *n* (%)			< 0.01
Acute coronary syndrome	53 (59)	34 (32)	
Chronic coronary artery disease	7 (8)	10 (10)	
Congestive heart failure	4 (4)	12 (11)	
Conduction disorders	16 (18)	8 (8)	
Structural disorders	7 (8)	7 (7)	
Unknown	3 (3)	34 (32)	
Therapeutic hypothermia	39 (43)	21 (20)	< 0.01
Intervention^c^, *n* (%)			
PCI	47 (52)	15 (14)	< 0.01
CABG	10 (11)	2 (2)	< 0.01
ICD	27 (30)	1 (1)	< 0.01
Pacemaker	8 (9)	3 (3)	0.11

*CPR* cardiopulmonary resuscitation, *AED* automated external defibrillator, *EMS* emergency medical services, *ROSC* return of spontaneous circulation, *ACLS* advanced cardiac life support, *PCI* percutaneous coronary intervention, *CABG* coronary artery bypass grafting, *ICD* implantable cardioverter defibrillator.

^a^Fisher’s exact or Chi-square test for categorical variables; Mann-Whitney *U* test for continuous variables.

^b^includes sinus rhythm, tachycardia, and bradycardia.

^c^Patients could have undergone more than one acute (sub) intervention.

### Pre-hospital characteristics of non-survivors in relation to survivors

Tables 1and 2 show the characteristics of the survivors and the non-survivors admitted to the emergency department. The non-survivors were relatively older compared with the survivors (70 [58–79] years) vs. 63 [55–72] years, *p* = 0.01), and chronic heart failure was present in 18 vs. 7 % (*p* = 0.02), respectively. An initial shockable rhythm was recorded in 69 non-survivors (67 %) vs. 81survivors (90 %), *p* < 0.01. Among the non-survivors < 50 % achieved ROSC at departure from the site and on arrival to the emergency department, while of the survivors nearly 100 % achieved ROSC at departure and arrival, *p* < 0.01, respectively. There was no statistical significance compared with survivors in terms of gender, witnessed arrest, bystander CPR, or use of AED.

### Diagnosis and treatment

Following admission to the emergency department, 50 (26 %) patients died. Table 2 shows the cardiac aetiology and treatment of OHCA. In 87 (45 %) of the patients, cardiac arrest was precipitated by acute coronary syndrome, 60 (31 %) received therapeutic hypothermia and 62 (32 %) patients underwent a PCI. Among the non-survivors, 21 (20 %) received therapeutic hypothermia, ACS was diagnosed in 34 (32 %) and 15 (14 %) underwent PCI procedure.

### Survival and discharge destination

Fifty-five patients died in hospital (Fig. 1) after an average stay of 8 days. Ninety (46.2 %) patients survived to hospital discharge after an average stay of 16 days following OHCA. After initial stabilisation and treatment, 17 patients were transferred to another hospital. Sixty-four (71 %) patients were discharged home and nine (10 %) to a nursing facility.

### Neurological outcome and follow-up

Table 3 illustrates the neurological outcome of the survivors at the time of hospital discharge and follow-up at the end of 1 month, 6 months and 12 months following OHCA. The neurological outcome (assessed by the modified Rankin Scale) at hospital discharge was available for 61 (68 %) patients. Accordingly, 50 % could carry out normal daily activities, while six (10 %) patients had moderately severe disability.

**Table 3:** The modified Rankin Scale scores at hospital discharge and follow-up at 1, 6 and 12 months

Modified Rankin Scale	Discharge *n* = 61	1 month *n* = 43	6 months *n* = 25	12 months *n* = 18
0	30 (50 %)	27 (63 %)	14 (56 %)	10 (56 %)
1	13 (21 %)	10 (23 %)	7 (28 %)	5 (28 %)
2	9 (15 %)	4 (10 %)	1 (4 %)	1 (6 %)
3	3 (5 %)	2 (5 %)	3 (12 %)	2 (11 %)
4	6 (10 %)	-	-	-
5	-	-	-	-

Modified Rankin Scale: 0—no symptoms, 1—no significant disability, 2—slight disability, 3—moderate disability, 4—moderately severe disability, 5—severe disability, 6—dead.

## Discussion

In our out-of-hospital cardiac arrest observational study, covering the Maastricht region, more than 46 % of the cardiac cohort admitted to the emergency department survived to hospital discharge. Evidently, shockable rhythm, ROSC on departure from site of arrest and arrival to the emergency department, shorter advanced cardiac life support duration, and post-arrest resuscitation care were the main findings for good survival outcome in the present study.

Recently, Boyce et al. reported a survival rate of 43 % in patients admitted to hospital due to cardiac and non-cardiac causes in the Leiden region [4]. In an earlier study reported from the Amsterdam region, survival rate to hospital discharge was 9 % [5].The varying survival outcome has been ascribed to regional variations following resuscitation efforts for OHCA and study design [1, 7, 8]. These described variations include but are not limited to population demographics, culture, competing illness, but also post-arrest care [16]. Despite all these regional variations, a trend towards a better outcome reflects improvements in the ‘chain of survival’ [6], as compared with the approximate 6 % reported from an earlier study in the 1990s, from the Maastricht region [17]. In nearly 75 % of the non-EMS witnessed cardiac arrests in the present study, a bystander provided basic CPR. It is known that bystander CPR maintains a state of shockable rhythm, which could double the chances of survival [5]. This finding is high compared to other Utstein reports [4–6], which is probably due to public awareness and alert systems in the region. Hence, early CPR determines the subsequent course of events. A state of shockable rhythm predicts better survival chances [18]. We observed a shockable rhythm to be most evident among the survivors (90 %). It is likely that an increased number of bystander CPRs in the acute phase of a cardiac event maintained a shockable rhythm. Furthermore, a correlation between shockable rhythm and ROSC has been described by Sasson and colleagues [2]. They reported that with a shockable initial rhythm, ROSC was achieved in 50 % of OHCA. In the ARREST study, Waalewijn et al. observed that 43 % of patients with a shockable rhythm achieved ROSC, while 13 % of patients with a non-shockable rhythm attained ROSC [5]. Therefore, the presence of a shockable rhythm might influence the duration of resuscitation and even the subsequent course of clinical management.

Recently, structured post-arrest care and its effect on the OHCA outcomes has been gathering attention, including the provision of therapeutic hypothermia, early cardiac intervention and neurological prognostication [1, 3, 18]. In our study 43 % of survivors were cooled down with therapeutic hypothermia comparable with 47 % of the survivors in the study by Boyce et al. [4]. Further, 43 % of the patients admitted to hospital underwent an acute and/or sub-acute intervention. In the recent OHCA study from the Netherlands, 86 % of patients admitted to the ICU or CCU underwent acute and/or sub-acute cardiac intervention [4]. The disparity might be due to the number of patients who died in our emergency department. A common understanding would be that patients with a reasonable chance to survive would receive further intervention. Potentially, early restoration of homeostasis with invasive CPR might serve as a bridge to definitive care in patient’s refractory to continued resuscitation. Following recovery, at hospital discharge, 50 % of documented cases in the present study had a favourable neurological outcome. Bloom et al. reported a favourable outcome in about 20 % of the ARREST study [18]. Plausibly, the presence of a shockable rhythm and early ROSC with consequent post-arrest care in eligible patients led to a better neurological outcome in our observational study.

One might wonder whether we could further optimise the chances of non-survivors towards better outcomes in the presented cohort. It is impossible to save every patient as some determinants remain unchanged, such as e.g. age and comorbidities. However, the modifiable determinant would be wider application of AED, thereby preventing a longer duration of no-flow (collapse to first defibrillation), which worsens the chances of survival by progressing into a non-shockable rhythm [8, 9]. Alternatively, the presence of a non-shockable rhythm among non-survivors (30 %) might have been due to failure of ICD, battery discharge following defibrillation or the duration of resucitation [19, 20]. Since there has been limited resucitative and post-arrest care options for non-shockable rhythm [8], one can argue that implementation of E-CPR, restoring circulation and oxygenation, might prevent a state of non-shockable rhythm [11, 12]. Therefore, it is tempting to speculate that a certain number of non-survivors in our cohort with survival determinants, such as witnessed arrest, received bystander CPR, or in shockable rhythm, would have likely benefited from invasive CPR in the event of unsustained ROSC, thereby, establishing a bridge to proven interventions.

E-CPR is considered in cardiac arrest patients with a brief no-flow period when the condition leading to cardiac arrest is reversible or amenable to heart transplantation [10]. Additionally, E-CPR needs to be implemented in experienced centres, where the technique can be rapidly initiated [11, 12, 21]. In a prospective, observational study by Chen et al. survival outcome improved drastically in patients receiving E-CPR [11]. An up to 42 % increase in survival rate was observed when E-CPR was implemented within 30 min of cardiac arrest. The implementation of E-CPR in a site like the emergency room would establish whole-body perfusion, providing a bridge to diagnosis and treatment, including controlled therapeutic hypothermia, cardiac intervention or heart transplant [11–13, 21, 22]. However, applying E-CPR may be futile in patients with previous cardiac comorbidities, i.e., chronic heart failure, structural heart disease or ICD failure. In the event of poor neurological prognosis, a decision for organ donation (based on advance directives and/or care provider’s decision) could improve the quality of organ donors as a secondary outcome [22]. Therefore, assuming that refractory cardiac arrest may be caused by a treatable condition, engaging all temporising techniques to facilitate further diagnostics and therapy might be prospectively applied in a select patient population; this might potentially improve survival rate and organ recovery.

Limitations of our analysis include the lack of neurological scores for all survivors, this information was not available at the time of discharge and follow-up. These patients were transferred to other regional hospitals or lost to follow-up.

## Conclusion

We reiterate that shockable rhythm, ROSC, and post-arrest care improves survival outcome. Potentially, initiating E-CPR in patients refractory to standard care on-scene or at the arrival to the emergency department might be a prospect for even better survival outcome.
